# Surgical Clinic

**Published:** 1866-09

**Authors:** R. G. Bogue

**Affiliations:** Attending Surgeon to the County Hospital


### HOSPITAL REPORTS.

*Surgical Clinic.* By R. G. Bogue, M. D., Attending Surgeon to the County Hospital. Reported by N. T. Quales, House Physician.

CASE I. STONE IN THE BLADDER—LITHECTASY—RECOVERY.

This little girl, three years of age, has been suffering from some urinary difficulty for more than a year—(for the last four months she has been worse)—being obliged to make water very often, passing but little at a time, always with more or less pain, and now complains most of the time of pain in the pubic region. She is fretful and irritable, and has the general appearance of suffering from some long-continued constitutional irritation.

From her general appearance and the local symptoms, it was suspected she might have stone in the bladder; and on introducing a catheter, to procure some urine for examination, a stone was reached. The little girl was etherized, and with a small, straight sound, the stone was felt very distinctly—the sharp click of the instrument was plainly heard. The stone seemed to be of some considerable size. The urine has been examined; it is acid, and loaded with crystals of uric acid and a large amount of urate of ammonia.

The operations for removal of the stone from the female are usually by crushing or by dilatation and incision of the urethra. In children the urethra does not dilate as largely in proportion as in adults. In this case the urethra has been dilated all that is possible by the use of compressed sponge tent, for several hours each day for the last three or four days.

[The little patient was etherized, the tent removed, and the mucous membrane of the urethra divided on either side of the median line inferiorly, by passing a straight, blunt-pointed tenotome into the urethra and cutting downward and a little outward. This allowed the introduction of a small pair of polypus forceps, a little curved at the end, with which, after a good deal of patient effort, the stone was seized and brought into the urethra, where it seemed too large to pass, and another incision was made in the mouth of the urethra upwards, and the stone removed. The bladder was washed out with tepid water, a cloth wet with cold water applied to the vulva, and potass acetat., grs. v., three times a day was prescribed. There was but little bleeding and very little pain afterward. She was put to bed and kept quiet. After about four hours she sat upon the chamber vessel and passed a few ounces of urine. Her recovery was rapid. She passed water regularly, and had incontinence only when the bladder became a good deal distended. She passed water not more than four times a day after the operation. There was very little soreness or tumefaction of the parts; all of which subsided in the course of three or four days. The stone was flattened and pointed, resembling in form an almond, weighing thirty-one grains, and composed of concentric layers of uric acid and urate of ammonia. She improved in general health and appearance, and was discharged, cured.]

### CASE II. RATTLESNAKE BITE—RECOVERY.

This patient has a poisoned wound of the hand, made by the bite of a rattlesnake. The diagnosis in these cases is always clear enough—from the circumstances and from the patient, the nature of the injury is readily ascertained. But as regards the treatment, much time has been spent by the profession, first, to find something to destroy the poison, and second, to prevent or counteract its effect. Various chemical agents have been used to destroy the poison. Dr. Mitchell, of Philadelphia, in his experiments, found no chemical agent that destroyed it when fresh from the snake. Professor Brainard, of this city, from a series of experiments, came to the conclusion that a solution of iodine, injected into the tissues about the wound, destroyed its effect. It is probable that if this be of any service, it must be used earlier than is common for a patient to be seen by a physician, for the poison is rapidly admitted into the circulation, where it would seem to be out of the reach of local remedies. It is most likely that little or nothing can be done to destroy the poison itself, but means must be used to prevent its fatal effect; which is, first, to destroy life by overwhelming the vital energies, and secondarily, by producing phlegmonous inflammations. The patient is to be kept alive until the poison can spend its force, or be eliminated. We must resort to free stimulation with alcohol, to which it might be of advantage to add carb. ammonia if there were excessive prostration. Heat should be applied to the extremities, and counter-irritants over the abdomen and thorax, if there is vomiting or difficulty in breathing.

Our patient is an Irishman, about 23 years old; by occupation a professional snake tamer; was admitted into the hospital, May 18th, about 11 o'clock in the evening, with a wound in the middle of the palm of the left hand, made by a rattlesnake five hours previously. The wound had been cauterized with nitrate of silver, and the man had taken rather freely of whisky. Condition, on admission, nearly comatose, surface cold, pulse not perceptible at the wrist, heart beat feeble, (55 per minute,) respiration 14, labored, constant vomiting, both hands and forearms swollen and very painful, glands in left axilla swollen and tender.

*Treatment.*—Tr. iodine applied freely to the hands and forearms, sinapisms to abdomen, heat to extremities. Stomach rejecting everything taken, six ounces of whisky were thrown into the rectum. Reaction soon began, and in the course of half an hour the pulse returned at the wrist, 80 per minute, respiration 18. Surface getting warmer, vomiting less frequent, and ceasing in the course of an hour, when about ℥ij. of whisky, with morphiæ sul. gr. $\frac{1}{2}$, were given and retained. He soon fell into a quiet sleep. No further medication during the night.

19th. Body warm; pulse about 80, moderately full; respiration 20, somewhat labored; moderate cough, with expectoration of bloody mucus; swelling of left limb extending up the arm, right only to the elbow; severe pain in left, moderate in right; about the wound were several large vesicles filled with very dark, bloody serum; has had several bloody passages from the bowels; no blood in the urine—(blood in the urine is common in these cases)—tongue swollen to nearly twice the normal size. He being very filthy, was given a warm bath. Tr. iodine again applied to the hands and arms, beef-tea for nourishment, and milk-punch given freely. Sodæ sulphis, gr. xv., every three hours; morphiæ sul. gr. ¼, every three or four hours, to quiet the pain.

20th. Much as yesterday, except less pain, and swelling subsiding in right limb; less cough, and expectoration not bloody; no blood in the passages from the bowels. Ordered flaxseed poultice to wound, and warm fomentations to the right hand and both arms. Other treatment continued, except the tr. iodine.

21st. To-day there is but little pain; swelling subsiding in both arms, he feels comparatively well every way, and is now out of danger.

In these cases the blood is poisoned, and it would seem to be disorganized, more or less depending upon the amount of the poison introduced; as it shows itself in the secretions from the bowels, kidneys and lungs. The iodine was used in the case not with the expectation of destroying any of the poison, but as I would use it in similar inflamed conditions of the skin and subcutaneous cellular tissue. The sulphite of soda is being used a good deal in cases of blood poisoning, and perhaps it may be of service in these cases—certainly it is orthodox in theory to use it.

[In the course of a few days the swelling and pain entirely subsided; the wound healed, and the man was discharged, cured, May 30th, 1866.]

